# Radiobiological modeling of two stereotactic body radiotherapy schedules in patients with stage I peripheral non-small cell lung cancer

**DOI:** 10.18632/oncotarget.9442

**Published:** 2016-05-18

**Authors:** Bao-tian Huang, Zhu Lin, Pei-xian Lin, Jia-yang Lu, Chuang-zhen Chen

**Affiliations:** ^1^ Department of Radiation Oncology, Cancer Hospital of Shantou University Medical College, Shantou 515031, China; ^2^ Department of Nosocomial Infection Management, The Second Affiliated Hospital of Shantou University Medical College, Shantou 515041, China

**Keywords:** radiobiological modeling, dose schedule, stereotactic body radiotherapy, non-small cell lung cancer

## Abstract

This study aims to compare the radiobiological response of two stereotactic body radiotherapy (SBRT) schedules for patients with stage I peripheral non-small cell lung cancer (NSCLC) using radiobiological modeling methods. Volumetric modulated arc therapy (VMAT)-based SBRT plans were designed using two dose schedules of 1 × 34 Gy (34 Gy in 1 fraction) and 4 × 12 Gy (48 Gy in 4 fractions) for 19 patients diagnosed with primary stage I NSCLC. Dose to the gross target volume (GTV), planning target volume (PTV), lung and chest wall (CW) were converted to biologically equivalent dose in 2 Gy fraction (EQD_2_) for comparison. Five different radiobiological models were employed to predict the tumor control probability (TCP) value. Three additional models were utilized to estimate the normal tissue complication probability (NTCP) value for the lung and the modified equivalent uniform dose (mEUD) value to the CW. Our result indicates that the 1 × 34 Gy dose schedule provided a higher EQD_2_ dose to the tumor, lung and CW. Radiobiological modeling revealed that the TCP value for the tumor, NTCP value for the lung and mEUD value for the CW were 7.4% (in absolute value), 7.2% (in absolute value) and 71.8% (in relative value) higher on average, respectively, using the 1 × 34 Gy dose schedule.

## INTRODUCTION

Stereotactic body radiation therapy (SBRT) is an effective and well-tolerated noninvasive treatment for patients with medically inoperable non-small cell lung cancer (NSCLC) [1–3]. Results from recent investigations demonstrate that SBRT treatment for early stage lung cancer can achieve outcomes comparable with surgical resection [4–6].

Although SBRT treatment for NSCLC has offered encouraging outcomes, investigations into the benefits of single and multiple dose schedules remain ongoing [7, 8]. To date, there are no clinical results comparing the outcomes between single and multiple fraction schedules; thus, the problem requires further investigation.

Radiobiological modeling is a method used to simulate the treatment outcome of the tumor and normal tissues using mathematical calculations with parameters generated from fitting the clinical trials. This method has the advantage of linking the dosimetric variation with radiobiological responses and was recently used to predict the feasibility of dose escalation for esophageal cancer and primary prostate cancer [9, 10].

Therefore, the study mainly aims to compare the dose response between single and multiple fraction SBRT dose schedules in terms of tumor control probability (TCP) and normal tissue complication probability (NTCP) using the method of radiobiological modeling. Given that a dose of 34 Gy in a single fraction [11] and 48 Gy in four fractions [12–14] stand as the maximal and the most widely used dose schedules, respectively, it is important to demonstrate their feasibility, safety, and efficacy. Thus, these two fractionation schemes are worthy of comparison in this study.

## RESULTS

### Patient characteristics

The patients' characteristics are listed in Table 1. In total, 14 of the patients suffered from T1 lesions and 5 of them suffered from T2 lesions. The median volume of gross target volume (GTV) and planning target volume (PTV) were 8.8 ±10.4 and 36.9 ±24.9 cm^3^, respectively.

**Table 1:** Characteristics of 19 patients with NSCLC undergoing SBRT

Patient	Gender	Age	Stage*	GTV (cm^3^)	PTV (cm^3^)
1	F	57	T1	0.9	8.1
2	M	35	T1	1.0	9.8
3	F	55	T1	2.1	20.2
4	M	71	T1	3.1	16.3
5	M	64	T1	3.3	23.0
6	M	62	T1	3.4	20.0
7	M	68	T1	3.6	27.6
8	F	59	T1	4.0	32.9
9	F	76	T1	4.2	23.2
10	M	68	T1	4.3	22.4
11	F	63	T1	4.6	39.9
12	F	72	T1	5.4	31.3
13	F	71	T1	6.9	28.7
14	F	62	T1	9.7	63.5
15	F	70	T2	10.3	39.5
16	M	70	T2	11.6	40.8
17	M	72	T2	21.0	71.0
18	M	77	T2	26.7	95.1
19	M	48	T2	41.6	87.9

Abbreviations: GTV = gross target volume; PTV = planning target volume;

M = Male; F = Female;

* *Note:*According to American Joint Committee on Cancer (AJCC), 7th edition.

### Dose comparison between the 1 × 34 Gy and 4 × 12 Gy dose schedule

**Figure 1:**
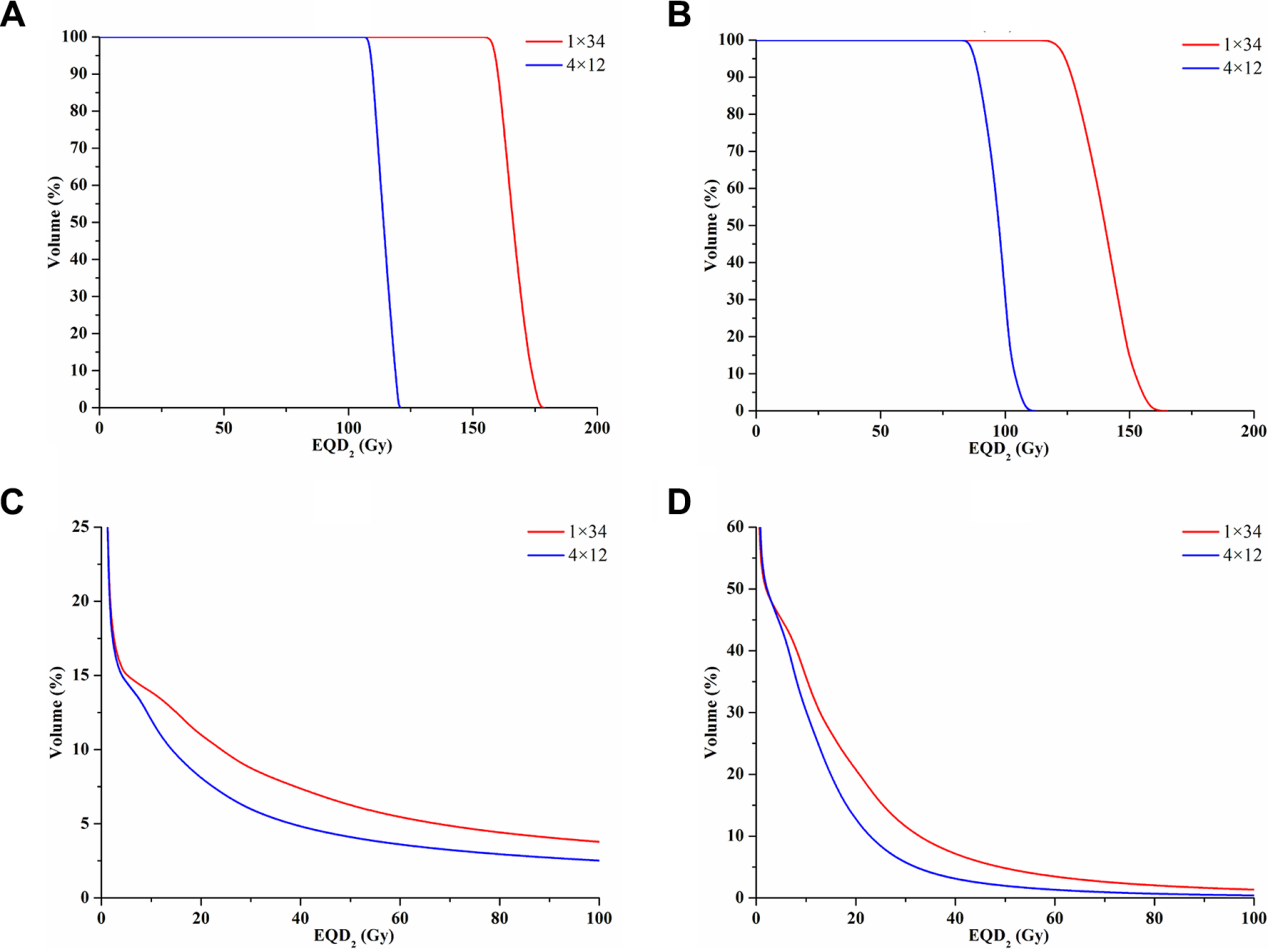
DVH of the GTV, PTV-GTV, lung and CW after EQD_2_ conversion (**A**) DVH of the GTV, (**B**) DVH of the PTV-GTV, (**C**) DVH of the lung, and (**D**) DVH of the CW. GTV = gross target volume; PTV = planning target volume; PTV-GTV = PTV minus GTV; CW = chest wall; EQD_2_ = biologically equivalent dose in 2 Gy fractions.

**Figure 2:**
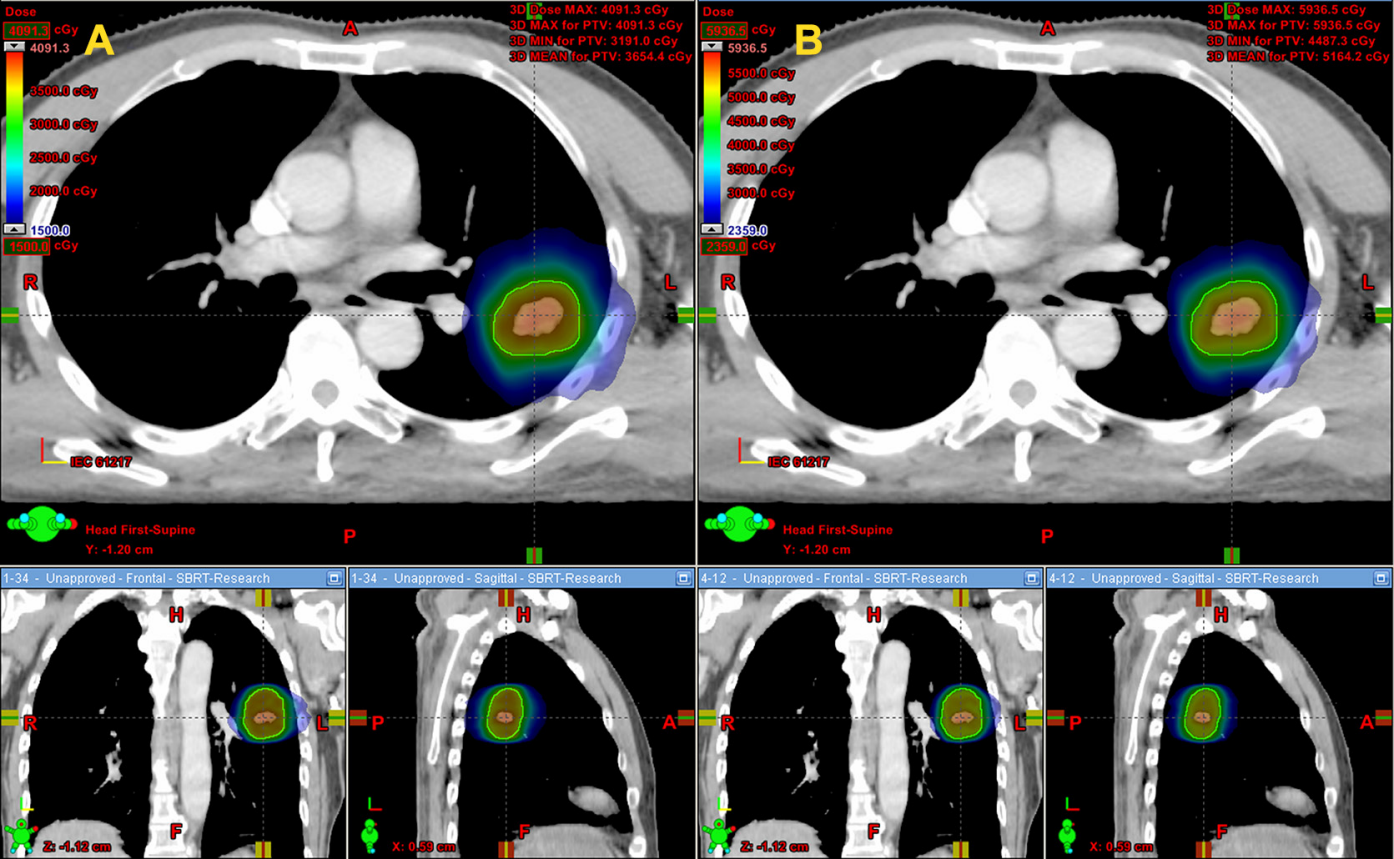
Dose distribution from the transversal, coronal and sagittal views between the two dose schedules The dose color wash slider was set at 15 Gy for the 1 × 34 Gy schedule and 23.59 Gy for the 4 × 12 Gy schedule (the same EQD_2_ dose). (**A**) Dose distribution of the 1 × 34 Gy schedule. (**B**) Dose distribution of the 4 × 12 Gy schedule.

Dose to the GTV, PTV-GTV (PTV minus GTV), lung and chest wall (CW) between the two dose schedules after conversion to biologically equivalent dose in 2 Gy fractions (EQD_2_) were listed in Table 2. All the dose comparisons were statistically significant with *p*-values < 0.05. Specifically, the 1 × 34 Gy schedule provided up to 47.1% and 44.1% higher dose on average for the GTV and PTV-GTV, respectively. Moreover, the 1 × 34 Gy schedule also added 3.1–7.9% more dose on average to V_10_–V_60_ of the CW. However, the dose delivered to the lung was much smaller, with V_5_–V_70_ increasing by 1.0–2.2%. The cumulative dose volume histogram (cDVH) of the GTV, PTV-GTV, lung and CW after EQD_2_ dose conversion is presented in Figure 1. Dose distributions from the transversal, coronal and sagittal views are presented in Figure 2.

**Table 2:** EQD_2_ dose comparison between 1 × 34 Gy and 4 × 12 Gy dose schedules

Structures	Parameter	1 × 34 Gy	4 × 12 Gy	*p*
GTV	D_mean_ (Gy)	167.6 ± 6.0	113.9 ± 3.5	0.000
PTV-GTV	D_mean_ (Gy)	140.9 ± 2.8	97.8 ± 1.7	0.000
Lung	V_5_ (%)	13.9 ± 5.0	12.9 ± 4.9	0.000
	V_10_ (%)	11.9 ± 4.7	10.4 ± 4.5	0.000
	V_20_ (%)	9.4 ± 4.0	7.2 ± 3.3	0.000
	V_30_ (%)	7.7 ± 3.4	5.5 ± 2.7	0.000
	V_40_ (%)	6.4 ± 3.0	4.4 ± 2.2	0.000
	V_50_ (%)	5.6 ± 2.7	3.6 ± 1.9	0.000
	V_60_ (%)	4.9 ± 2.4	3.1 ± 1.6	0.000
	V_70_ (%)	4.4 ± 2.2	2.7 ± 1.4	0.000
	D_mean_ (Gy)	12.4 ± 7.3	7.0 ± 3.6	0.000
CW	V_10_ (%)	39.2 ± 12.1	34.7 ± 12.1	0.000
	V_20_ (%)	25.6 ± 11.1	17.7 ± 8.6	0.000
	V_30_ (%)	16.5 ± 8.0	8.6 ± 5.2	0.000
	V_40_ (%)	10.7 ± 5.8	4.6 ± 3.8	0.000
	V_50_ (%)	7.1 ± 4.7	2.8 ± 2.7	0.000
	V_60_ (%)	5.0 ± 3.9	1.9 ± 2.0	0.000
	D_mean_ (Gy)	24.8 ± 10.4	15.3 ± 5.6	0.000

Abbreviations: GTV = gross target volume; PTV = planning target volume; PTV-GTV = PTV minus GTV. CW = chest wall; D_mean_ = mean dose; V_x_ was the volume of the organ receiving a dose of x Gy or more.

### Dose response between the 1 × 34 Gy and 4 × 12 Gy dose schedule

By radiobiological modeling, we found that five TCP and two lung complication predicting models yielded similar results. Radiobiological modeling revealed that TCP value for the tumor and NTCP value for the lung were 7.4% and 7.2% higher (in absolute value), respectively, on average with the 1 × 34 Gy schedule compared with the 4 × 12 Gy schedule. The modified equivalent uniform dose (mEUD) value to the CW was also increased by 71.8% in relative value. Detailed information of TCP prediction in the five models is presented in Table 3. Data regarding the NTCP for the lung and mEUD value to the CW between the two dose schedules are provided in Table 4.

**Table 3:** TCP value between 1 × 34 Gy and 4 × 12 Gy dose schedule in five models

Parameter	1 × 34 Gy	4 × 12 Gy	*p*
Mar (%)	98.3 ± 0.4	85.3 ± 2.5	0.000
Fen (%)*	95.8 ± 1.3	90.9 ± 3.5	0.000
WN (%)	94.3 ± 0.7	85.5 ± 2.0	0.000
EUD (%)	98.1 ± 0.2	93.4 ± 0.7	0.000
Nitin (%)	98.1 ± 0.7	92.6 ± 2.4	0.000
Median (%)	96.9 ± 1.7	89.5 ± 4.2	0.000

Abbreviations: Mar = Martel model; Fen = Fenwick model; WN = Webb-Nahum model; EUD = equivalent uniform dose model; Nitin = Nitin model.

* *Note:* Indicates Fenwick model for TCP prediction.

**Table 4:** NTCP value for the lung and mEUD value for the CW between 1 × 34 Gy and 4 × 12 Gy dose schedules

Parameter	1 × 34 Gy	4 × 12 Gy	*p*
Lung			
LKB (%)	13.6 ± 10.2	5.8 ± 3.0	0.000
Fen (%)*	11.6 ± 8.7	4.9 ± 2.5	0.000
Median (%)	12.6 ± 9.4	5.4 ± 2.8	0.000
CW			
mEUD (%)	175.1 ± 89.1	101.9 ± 48.9	0.000

Abbreviations: LKB = Lyman-Kutcher-Burman (LKB) model; Fen = Fenwick model; mEUD = modified equivalent uniform dose model; CW = chest wall.

* *Note:* Indicates Fenwick model for NTCP estimation to the lung.

## DISCUSSION

Our analysis of the dose response for stage I NSCLC using radiobiological modeling revealed that the TCP value for the tumor was 7.4% higher with the 1 × 34 Gy dose schedule. In addition, the NTCP value for the lung and mEUD value to the CW were increased by 7.2% in absolute value and 71.8% in relative value on average, respectively, compared with the 4 × 12 Gy schedule. To our knowledge, our study is the first to use different radiobiological models to predict TCP and NTCP data in single and multiple fraction dose schedules. We believe that our results can provide useful information for clinical SBRT treatment of lung lesion.

The incidence of radiation pneumonitis (RP) ranges from 10% to 20.3% [15–17], and the median intervals to first graphical appearance were 4.2 and 2.5 months for grade 1 and grade 2–3 RP, respectively [18]. Similarly, the incidence of CW toxicity ranged from 8.3% to 32.8% [19–23] and frequently occurred greater than 6 months after the completion of therapy [23]. As the RP and CW pain are common radiotherapy-induced side effects for peripheral NSCLC patients undergoing SBRT, our study mainly focuses on predicting the complication probability of the lung and CW between the two dose schedules.

The 1 × 34 Gy and 4 × 12 Gy dose schedules have been widely used in SBRT treatment for lung cancer and many publications have reported their feasibility, safety and efficacy in clinical treatment. Hara et al reported that irradiation doses of ≥ 30 Gy resulted in 1-year local progression-free rates (LPFRs) of 93% in fifty tumors and Grade 3 respiratory symptoms were only noted in 1 patient [11]. Videtic et al observed 1-year local control of 86.2% and no grade 3 or higher toxicity with the 1 × 34 Gy schedule [24]. Nagata et al observed that 98% of 45 tumors were locally controlled during the follow-up period, and no pulmonary complications greater than Grade 3 were noted with the 4 ×12 Gy scheme [12]. Kelley et al reported 81.8% local control at one year for the entire cohort and no grade 3 or greater toxicity adverse events were observed with the 4 × 12 Gy scheme for treatment of medically inoperable NSCLC patients [13]. The mentioned outcomes demonstrate comparable local control and incidences of adverse events between the two dose schedules. However, our finding that the TCP for the tumor and the risk of radiation-induced complication were higher with the 1 × 34 Gy schedule slightly differs from the results of these studies. Given that the study was comparing the TCP and NTCP using two schedules of prescription dose, it is not suitable to compare this study using radiobiological modeling with the clinical data of the previous study. How to better fit to clinical data with radiobiological models will be a subject of our future work.

The repopulation and reoxygenation in tumor cells are two problematic factors that should be taken into account during the radiobiological modeling of single and multiple fraction schedules. Single-fraction SBRT is a promising modality that has the potential to circumvent the problem of repopulation, which can occur during conventional, fractionated radiation therapy [11]. The impact of tumor cell repopulation could be ignored when accelerated repopulation was observed in less than 21 days [25], as noted in lung SBRT treatment. Regarding tumor reoxygenation, multiple fraction schedules are more beneficial for hypoxic tumors, as noted in lung tumors. However, the impact of inter-fraction fast reoxygenation of the hypoxic cells remains unclear, particularly in small fraction SBRT treatment. The recent investigation demonstrated a reduction in D_50_ from 53.3 Gy for 2 fractions to 52.7 Gy for 5 fractions for hypofractionated treatments employing large doses per fraction due to a synergistic effect between intra-fraction repair and inter-fraction fast reoxygenation of the hypoxic cells [26]. The results indicated a slight impact of tumor cells' reoxygenation in less than 5 fractions of treatment. In summary, we believe the impact of repopulation and reoxygenation of the tumor cells on radiobiological modeling is negligible because our study only includes 1 and 4 fraction dose schedules.

The EQD_2_ conversion in the present study was performed using the linear-quadratic (LQ) model that is derived from biological considerations of how cells could be killed by ionizing radiation [27]. The suitability of the LQ model for high doses has been intensely debated in recent years. Recently, increasing clinical evidence has confirmed the accuracy of LQ-based tumor control and normal tissue predicting models. Guckenberger et al suggested that the traditional LQ formalism is accurately modeled for stage I NSCLC patients undergoing fractionated SBRT based on 395 patients from 13 German and Austrian centers [28]. Shuryak et al also found that LQ-based TCP models can provide significantly better fits to single-fraction local control data for SBRT treatment of NSCLC compared with other models requiring extra terms at high doses [29]. Borst et al demonstrated that the LQ model was valid for estimating the toxicity probabilities of RP [30]. According to two clinical studies, we believe that the dose conversion in our study utilizing the LQ model is reliable.

The results of our analysis are dependent on the choice of radiobiological models and parameters used. Therefore, we used a series of models from the literature to confirm our result. Five TCP predicting models were used in the present study and the radiobiological parameters of four models were derived from clinical data. Particularly, the Nitin model was generated by retrospectively analyzing 504 NSCLC tumors treated with a variety of SBRT schedules [31]. Interestingly, different radiobiological models predicted similar trends, irrespective of the TCP or NTCP prediction, suggesting their feasibility in predicting the radiobiological response.

Although our study has predicted the dose response of 1 × 34 Gy and 4 × 12 Gy schedules, our study has some limitations. (1) Our analysis is mainly based on radiobiological modeling and the results should be confirmed in large prospective, randomized, controlled clinical trials. (2) We ignored the radiation-induced rib fracture in lung SBRT. Although several studies reported that the tumor-chest wall distance is a risk factor for rib fracture [32], no radiobiological model has been proposed to predict the incidence of this condition; thus, we could not easily evaluate rib fracture during the modeling.

In conclusion, radiobiological modeling analysis demonstrates that the 1 × 34 Gy dose schedule achieves 7.4% higher TCP prediction but also increases radiation-induced lung and CW complication probabilities by 7.2% (absolute value) and 71.8% (relative value), respectively, compared with the 4 × 12 schedule.

## MATERIALS AND METHODS

### Ethics statement

The protocol was approved by the Ethics Committee of Cancer Hospital of Shantou University Medical College. Given that this is not a treatment-based study, our institutional review board waived the need for written informed consent from the participants. However, patient information was anonymous to protect their confidentiality.

### Patients selection

Nineteen patients previously diagnosed with peripheral stage I primary NSCLC and treated with radiotherapy were used in this study. A peripheral lesion was defined as the tumor > 2 cm in all directions around the proximal bronchial tree according to the definition in Radiation Therapy Oncology Group (RTOG) 0915 report [33]. Meanwhile, the tumor must be beyond 1 cm of major vessels, esophagus, heart, trachea, pericardium, brachial plexus and vertebral bodies. Inclusion criteria in the study included (1) histological confirmation of NSCLC prior to treatment, (2) stage T1N0M0 or T2N0M0 (maximal diameter ≤ 5 cm in any direction), (3) age > 18 years old.

### Computed tomography (CT) scanning

The patients were simulated on a supine position with a vacuum bag (Medtec Medical, Inc. Buffalo Grove, IL) or a thermoplastic mask (Guangzhou Klarity Medical & Equipment Co., Ltd, Guangzhou, China) restricting system. All of the patients underwent respiratory-correlated four dimensional computed tomography (4DCT) scans using Brilliance CT with Big Bore (Cleveland, OH, USA). CT images were obtained at a 3-mm slice thickness during scanning. CT images were then transferred to an Eclipse treatment planning system (Version 10.0, Varian Medical System, Inc., Palo Alto, CA) for target delineation, organs at risk (OARs) contouring and treatment planning.

### Delineation of target volume and OARs

For target contouring, the internal target volume (ITV) was defined as the integration of the GTV on 10 phases of the 4DCT in the pulmonary windows. To account for set-up uncertainties and mechanical tolerance, a PTV was created by adding a uniform 5-mm margin expansion to the ITV. As to normal tissue contouring, the entire lung was limited to the air-inflated lung parenchyma, and the GTV and trachea/ipsilateral bronchus were excluded according to the RTOG 0915 report [33]. The CW was segmented from the corrected lung edges with a 20-mm expansion in the lateral, anterior, and posterior directions, excluding the lung volume and the mediastinal soft tissue [19, 34, 35]. If the 20-mm expansion extended outside the body, the contour extended only as far as the external patient surface [35]. To avoid cumbersome contouring of the entire CW, we defined the CW within a 30-mm limit in the head-to-feet direction from the PTV [34].

### Treatment planning

Two dose schedules of 1 × 34 Gy and 4 × 12 Gy were prescribed. The treatment plans were designed using the Eclipse treatment planning system and conducted on the averaged 4DCT. Treatment plans were designed using the volumetric modulated arc therapy (VMAT) technique. All plans were designed on a TrueBeam Linac with a 6 MV flattening filter free (FFF) photon beam (maximum dose rate of 1400 MU/min). Plans were created using dual partial arcs to prevent irradiation from entering the contralateral lung. The collimator angles for all plans were set to 30° and 330° to minimize the contribution of the tongue-and-groove effect to the dose. Optimization was performed using the progressive resolution optimizer (PRO_10028) algorithm implemented in Eclipse 10.0. Dose calculation was performed using the anisotropic analytical algorithm (AAA_10028) with a grid resolution of 1 mm, considering the heterogeneity correction. The dose was normalized to 95% of the PTV receiving the prescribed dose. Prescription constraints, intermediate dose spillage and critical organ dose-volume limits in the two dose schedules were based on the criterion of the RTOG 0915 protocol [33]. Because RTOG 0915 report demands rapid dose fall-off beyond the target, the dose to the GTV was always greater than 120% of the prescribed dose.

### Dosimetric evaluation of the two dose schedules

For clarification, all doses mentioned in the article were converted to the EQD_2_ dose using the LQ model. The target was divided into two substructures of GTV and PTV-GTV for comparison. For GTV and PTV-GTV, the evaluating parameter was the mean dose. For the lung and CW, the analysis included the mean dose and a set of dose volume histogram (DVH)-based values. cDVH for the target and OARs were reconstructed from the individual DVH. These histograms were obtained by averaging the corresponding volumes for each dose bin of 0.05 Gy. Dose distributions from the transversal, coronal and sagittal views between the two dose schedules were acquired at 15 Gy and 23.59 Gy (with the same EQD_2_ dose) for the 1 × 34 Gy and 4 × 12 Gy schedules, respectively.

### Radiobiological modeling

**Figure 3:**
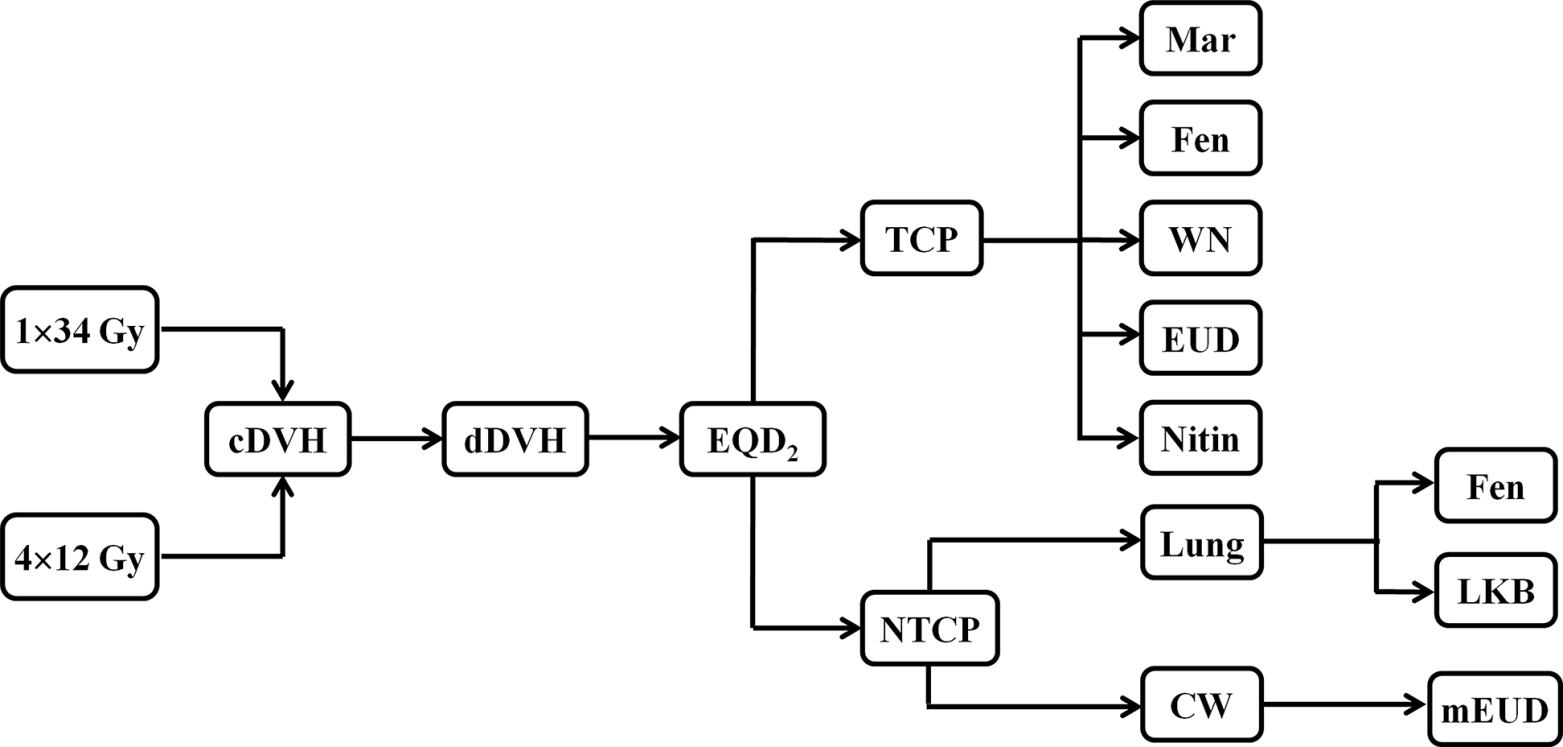
Flow chart of the radiobiological modeling cDVH = cumulative dose volume histogram; dDVH = differential dose volume histogram; EQD_2_ = biologically equivalent dose in 2 Gy fractions; TCP = tumor control probability; NTCP = normal tissue complication probability; Mar = Martel model; Fen = Fenwick model; WN = Webb-Nahum model; EUD = equivalent uniform dose model; Nitin = Nitin model; LKB = Lyman-Kutcher-Burman (LKB) model; mEUD = modified equivalent uniform dose model; CW = chest wall.

Both the TCP and NTCP were calculated using in-house developed programs using MATLAB 7.0 (MathWorks, USA). The TCP was calculated using five different radiobiological models, including the Martel model [36], Fenwick model [37], Webb-Nahum model [38], equivalent uniform dose (EUD) model [39] and Nitin model [31]. We utilized the EUD-based Lyman-Kutcher-Burman (LKB) model [40] and Fenwick model [37] to estimate the NTCP for the lung. Radiation-induced CW toxicities were predicted for the 100-cc highest dose region using the mEUD model with moderate weighting [20]. A flow chart of the radiobiological modeling is presented in Figure 3. A detailed modeling procedure is described as follows: (1) cDVH statistics of GTV, lung and CW were imported into MATLAB software at a resolution of 0.05 Gy. (2) Then, the cDVH statistics were converted to the differential dose volume histogram (dDVH) according to the method of Hiram [39]. (3) To reduce the effects of heterogeneity in the two dose schedules, the program converted the dose in each volume element to an EQD_2_ dose using the formula reported by other publications [41, 42]. (4) Finally, the main program of different calculation models and the TCP and NTCP values were automatically calculated. An α/β value of 10 Gy was assigned for the tumor during the EQD_2_ conversion [43, 44]. α/β values of 1.3 Gy and 3 Gy were assigned to estimate the NTCP for the lung and the mEUD value to the CW, respectively [25, 45]. Radiobiological parameters in the Martel, Fenwick, Nitin and mEUD models were obtained from the original work. Particularly, α_m_ and σ_a_ values in the Webb-Nahum model were 0.30 and 0.1, respectively, by averaging 10 different histological sub-types of human lung cancer cell lines from Carmichael's report [46]. Cell density, ρ was equal to 10^8^ according to Lindblom's work [47]. TCD_50_ and γ_50_ parameters in the EUD model were obtained from Okunieff's work [48], and α was 0.30 according to Carmichael's report [46]. TD_50_, n and m in the LKB model were obtained from Seppenwoolde's result [49]. The larger the TCP, NTCP and mEUD values, the higher control rate for the tumor or higher incidence rate of complications in the lung and CW.

### Statistical analysis

Median values and standard deviation are reported in the study. The Statistical Package for Social Sciences (SPSS, version 19.0, Chicago, IL) was used for statistical analysis. Comparison of the DVH-based parameters was performed using paired, two-tailed Student's *t*-test. A Wilcoxon matched-pair signed-rank test was alternatively used when the data did not follow a normal distribution. The results were considered statistically significant when *p*-values < 0.05.
